# Surgical Repair of an Infrarenal Aortic Aneurysm in an Infant With Tuberous Sclerosis

**DOI:** 10.7759/cureus.105777

**Published:** 2026-03-24

**Authors:** Aishwarya Pal, Simha Swaraj Sirivela, Shweta Mallick, Sidharth Viswanathan

**Affiliations:** 1 Department of Gastrointestinal Surgery and Solid Organ Transplantation, Amrita Institute of Medical Sciences, Kochi, IND; 2 Department of Vascular Surgery, Amrita Institute of Medical Sciences, Kochi, IND

**Keywords:** abdominal aortic aneurysm, aortic aneurysm repair, expanded polytetrafluoroethylene (eptfe), genetic vascular disorders, pediatric vascular surgery, prosthetic graft thrombosis, prosthetic vascular graft, tsc2 gene, tuberous sclerosis complex (tsc), vascular surgery

## Abstract

Pediatric abdominal aortic aneurysms are exceedingly rare, particularly in infants, and pose unique diagnostic and surgical challenges. We present a case of an infant diagnosed antenatally with intracardiac rhabdomyomas who later presented with infantile spasms. Neuroimaging and genetic testing confirmed tuberous sclerosis complex with a pathogenic de novo TSC2 mutation. Tuberous sclerosis complex is associated with dysregulation of the mammalian target of rapamycin (mTOR) signaling pathway, leading to abnormal vascular smooth muscle proliferation, structural vessel wall weakness, and predisposition to aneurysm formation. The increased cellular proliferation and altered vascular biology may also contribute to a prothrombotic milieu, particularly in the postoperative setting following vascular reconstruction. Systemic evaluation of the patient revealed a large fusiform infrarenal abdominal aortic aneurysm, following which the patient underwent elective open aneurysm repair using an 8-mm expanded polytetrafluoroethylene graft. The patient developed early postoperative graft thrombosis, necessitating emergency graft revision. Following re-exploration, satisfactory distal perfusion was achieved, and the postoperative recovery was uneventful.

This case highlights the importance of routine vascular screening in patients with tuberous sclerosis complex, underscores the underlying pathophysiology of vascular involvement, and outlines the surgical challenges associated with managing abdominal aortic aneurysms in infancy.

## Introduction

Aortic aneurysms in childhood are exceptionally rare, with an estimated prevalence of fewer than one in 100,000 children [1]. Pediatric abdominal aortic aneurysms account for less than 2% of all aortic aneurysms and are most commonly secondary to infection, trauma, or underlying genetic and connective tissue disorders [2]. Tuberous sclerosis complex is an autosomal dominant multisystem disorder affecting approximately one in 6,000-10,000 live births and is characterized by hamartomatous involvement of multiple organs [3]. Vascular manifestations in tuberous sclerosis are uncommon but increasingly recognized and are thought to result from dysregulation of the mammalian target of rapamycin (mTOR) pathway, leading to abnormal vascular smooth muscle proliferation, disruption of normal vessel wall architecture, and a predisposition to aneurysm formation [4]. In addition, altered vascular integrity and endothelial dysfunction may contribute to an increased risk of thrombosis, particularly in the postoperative setting following vascular reconstruction.

This case underscores the importance of considering vascular involvement in patients with tuberous sclerosis, demonstrates the feasibility of open surgical repair in infancy, and highlights postoperative challenges such as early graft thrombosis.

## Case presentation

A six-month-old infant had been diagnosed antenatally with cardiac rhabdomyomas during routine obstetric ultrasonography. The child was born at term via an uncomplicated vaginal delivery to non-consanguineous parents, with a normal birth weight. Hypomelanotic macules were noted during the neonatal period. During infancy, the patient developed infantile spasms and exhibited delayed developmental milestones, including poor head control and limited interaction with objects. Further evaluation led to a diagnosis of tuberous sclerosis complex. As part of systemic screening, an asymptomatic infrarenal abdominal aortic aneurysm was identified.

**Figure 1:**
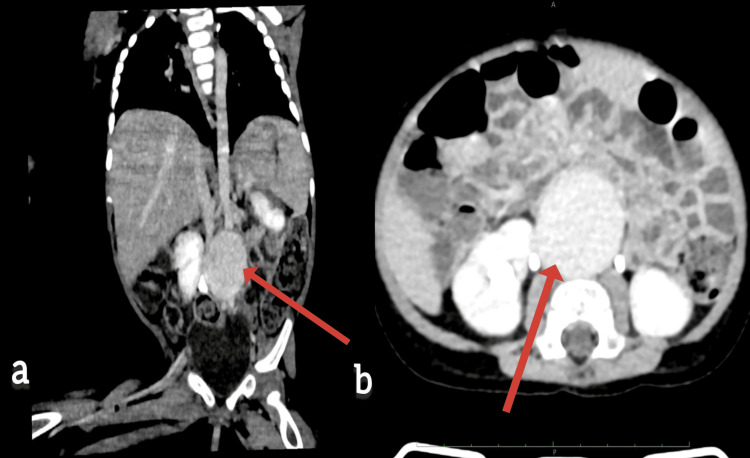
Contrast-enhanced computed tomography (CECT) showing an infrarenal aneurysm.

Electroencephalography initially showed no definitive epileptiform discharges, while magnetic resonance imaging of the brain demonstrated multiple cortical tubers and subependymal nodules consistent with tuberous sclerosis complex. Genetic analysis revealed a pathogenic de novo nonsense mutation in the TSC2 gene. Transthoracic echocardiography identified multiple intramural cardiac rhabdomyomas involving the left ventricular posterior wall, interventricular septum, and right ventricular free wall. Abdominal ultrasonography revealed an infrarenal abdominal aortic aneurysm extending to the aortic bifurcation, which was further characterized on contrast-enhanced computed tomography as a fusiform aneurysm measuring approximately 5 × 3.3 cm (anteroposterior × transverse), located 1 cm distal to the renal arteries (Figure 1).

**Figure 2:**
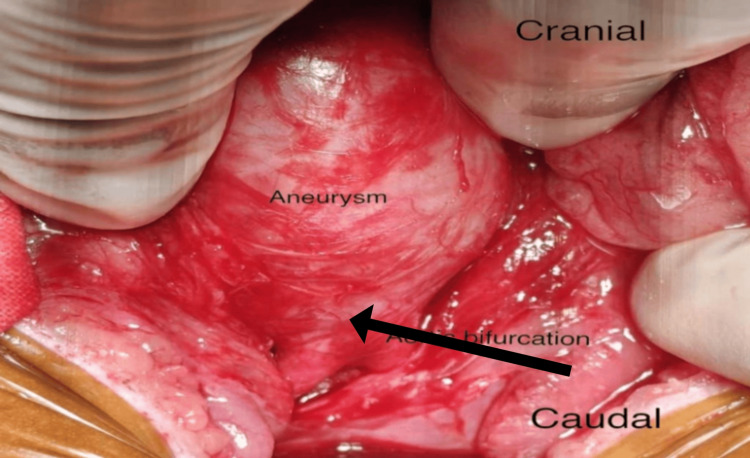
Intraoperative image showing a fusiform abdominal aortic aneurysm ending at the aortic bifurcation.

**Figure 3:**
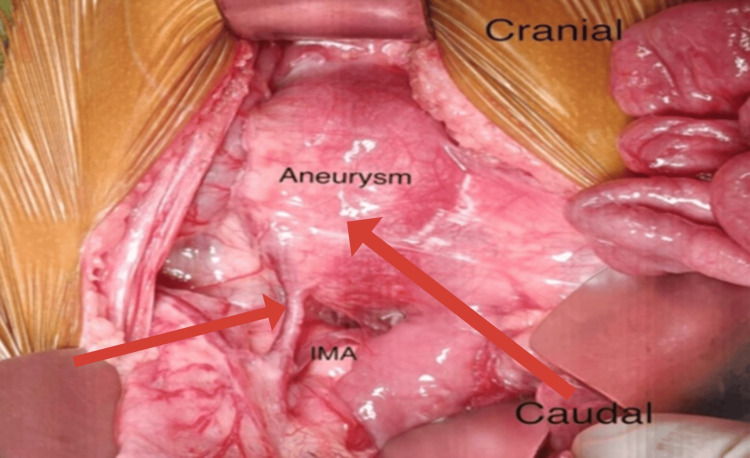
Infrarenal aneurysm of size 4 cm, with the inferior mesenteric artery (IMA) arising from the aneurysmal sac.

The patient subsequently underwent elective open repair of the infrarenal abdominal aortic aneurysm via a transperitoneal approach, with a transverse supraumbilical incision. Intraoperatively, a fusiform true aneurysm of the infrarenal aorta measuring approximately 4 cm in diameter was identified, with a 1 cm infrarenal neck. The aneurysm extended distally up to the aortic bifurcation (Figure 2), while both iliac arteries were noted to be normal. The inferior mesenteric artery was patent and was seen arising from the aneurysmal sac (Figure 3).

The infracolic compartment was approached by retracting the transverse colon cephalad and the small bowel to the right. The posterior peritoneum was then opened over the aneurysmal sac, extending up to the duodenojejunal flexure and the inferior mesenteric vein. Proximally, the left renal vein was identified, and distal dissection was continued to expose the infrarenal aortic neck. Both common iliac arteries were carefully dissected and prepared for cross-clamping. The inferior mesenteric artery was identified at its origin, dissected, and ligated. Systemic heparinization was administered at a dose of 1 mg/kg, following which the infrarenal aorta and bilateral iliac arteries were clamped.

**Figure 4:**
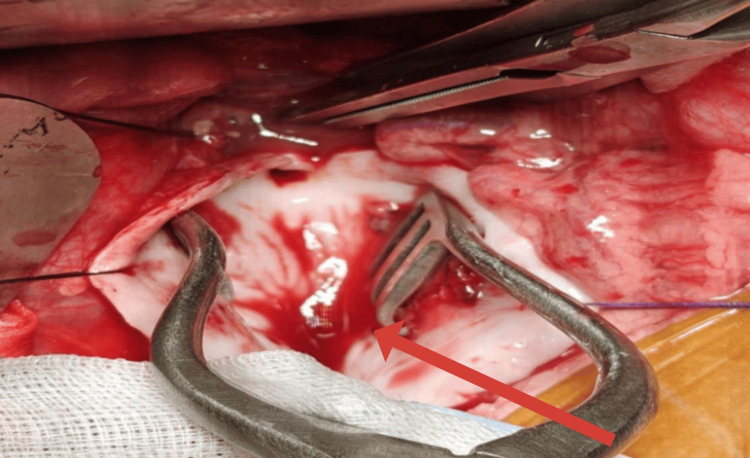
Intraoperative image showing the opened aneurysmal sac and proximal neck of the aneurysm visualized following clamping.

**Figure 5:**
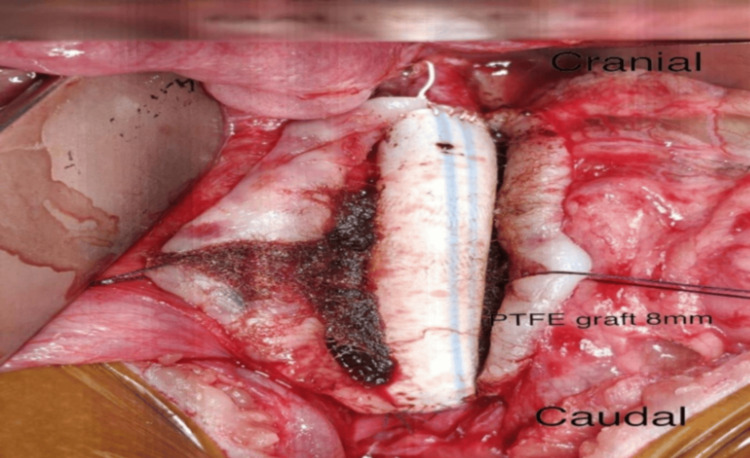
Intraoperative image showing an 8 mm polytetrafluoroethylene (PTFE) graft used for aneurysmal repair.

The aneurysm sac was opened vertically (Figure 4), and T-shaped incisions were made at the aortic neck and bifurcation. Minimal mural thrombus was observed. A few patent lumbar arteries were identified posteriorly with active back-bleeding and were oversewn using 6-0 polypropylene sutures. An 8-mm expanded polytetrafluoroethylene (ePTFE) graft was selected and trimmed to the appropriate length. Proximal end-to-end anastomosis was performed using CV-5 polytetrafluoroethylene sutures in a continuous fashion. Following completion of the proximal anastomosis, the clamp was shifted onto the graft. The distal end-to-end anastomosis was then completed using 6-0 polypropylene sutures in a continuous manner. After adequate de-airing and flushing, distal perfusion was restored sequentially. Additional sutures were placed as required to ensure complete hemostasis (Figure 5).

A large lymphatic vessel overlying the proximal aorta was identified and double-clipped to prevent a chyle leak, following which adequate Doppler signals were confirmed in both iliac arteries. The aneurysm sac was then approximated over the graft using 3-0 polyglactin sutures, and a small portion of the sac was excised and sent for histopathological examination, while the remaining abdominal viscera appeared normal. Within hours postoperatively, Doppler examination demonstrated the absence of graft flow in the femoral arteries, suggestive of acute graft occlusion. Emergency re-exploration revealed acute graft thrombosis without evidence of technical failure at the anastomotic sites, and the graft was subsequently revised using a similar 8-mm expanded polytetrafluoroethylene conduit. The distal iliac arteries were flushed with heparinized saline, and adequate thrombus clearance was confirmed, following which satisfactory bilateral femoral and distal Doppler signals were obtained.

The subsequent postoperative course was uneventful, with bilateral femoral artery pulses remaining palpable and good distal perfusion maintained. The child was initiated on continuous heparin infusion with activated partial thromboplastin time (aPTT) monitoring and was discharged in stable condition, with plans for regular clinical and imaging surveillance to monitor graft patency and growth-related vascular changes.

## Discussion

Abdominal aortic aneurysms in the pediatric population are exceedingly rare, particularly in neonates and infants, and differ significantly from adult aneurysms in terms of etiology, natural history, and management [1,2]. Unlike adults, where atherosclerosis is the predominant cause, pediatric aneurysms are more commonly associated with infection, trauma, vasculitis, connective tissue disorders, and genetic syndromes [2,5]. Acquired causes include bacterial or fungal infections, vasculitides such as Takayasu arteritis, polyarteritis nodosa, and Kawasaki disease, as well as iatrogenic injury related to umbilical artery catheterization [5,6]. These processes lead to inflammatory destruction of the vascular media and progressive weakening of the aortic wall.

Congenital abdominal aortic aneurysms represent an even rarer subset and may arise from developmental abnormalities of the vascular wall during embryogenesis [7]. Structural defects in the aortic media can result in focal weakness and subsequent aneurysmal dilatation. Vascular involvement in tuberous sclerosis complex is attributed to mutations in the TSC1 or TSC2 genes, which result in loss of inhibition of the mammalian target of rapamycin (mTOR) signaling pathway [3]. This leads to unchecked cellular growth and proliferation, particularly affecting vascular smooth muscle cells and perivascular epithelioid cells. Consequently, there is abnormal smooth muscle proliferation, disorganized vessel wall architecture, and fragmentation of elastic fibers within the tunica media, all of which contribute to structural weakening of the vessel wall. In addition, endothelial dysfunction and altered extracellular matrix remodeling further impair vascular integrity. These changes predispose to the formation of aneurysms, stenotic lesions, and vascular dysplasia in various arterial beds. The increased cellular proliferation and altered vascular biology may also contribute to a prothrombotic milieu, particularly in the postoperative setting following vascular reconstruction, thereby increasing the risk of early graft thrombosis despite technically adequate repair.

Mutations in the TSC2 gene, as identified in our patient, are associated with a more severe phenotypic expression compared to TSC1 mutations, likely due to greater loss of inhibition of the mTOR signaling pathway, resulting in enhanced cellular proliferation, aberrant vascular smooth muscle cell differentiation, and structural vessel wall abnormalities that may predispose to vascular lesions. Although classically associated with neurological, renal, cardiac, and dermatologic manifestations, vascular involvement has also been reported, with aneurysms of the aorta and other arteries described in affected patients [4,8].

Management of pediatric abdominal aortic aneurysms remains challenging because standardized guidelines regarding the timing of intervention and size thresholds are lacking. Owing to the unpredictable natural history and potential risk of rupture, early surgical intervention is generally recommended once the diagnosis is established [9], even in asymptomatic patients, to prevent rapid aneurysmal expansion, thrombus formation with distal embolization, progressive luminal compromise, and catastrophic complications such as dissection or rupture, which may occur at smaller diameters compared to adult populations. Endovascular repair is rarely feasible in neonates and infants because of small vessel diameter and the absence of appropriately sized devices; therefore, open surgical repair remains the primary treatment modality in this age group [9]. Surgical options include aneurysmorrhaphy, patch repair, and interposition grafting, with prosthetic grafts such as expanded polytetrafluoroethylene demonstrating acceptable long-term outcomes in pediatric vascular surgery [10].

Graft selection in infants presents unique challenges due to the mismatch between the small native aortic diameter and the available graft sizes, and relatively larger grafts may be required to maintain patency while accommodating future somatic growth [11]. Early postoperative graft thrombosis, although uncommon, is a recognized complication and requires prompt recognition and intervention to restore distal perfusion. Recent reports have also described abdominal aortic aneurysms associated with tuberous sclerosis presenting later in childhood or adolescence, highlighting the variable spectrum of vascular involvement in this condition [11].

Early postoperative graft thrombosis, as observed in this patient, is an uncommon but serious complication. In patients with tuberous sclerosis complex, an underlying hypercoagulable state, abnormal vascular smooth muscle proliferation, or intrinsic endothelial dysfunction may contribute to thrombosis despite technically satisfactory anastomoses. This underscores the importance of meticulous surgical technique, adequate intraoperative anticoagulation, early postoperative Doppler surveillance, and prompt re-intervention when indicated. A multidisciplinary approach involving pediatric surgeons, vascular surgeons, neurologists, cardiologists, intensivists, and geneticists is essential to optimize both short- and long-term outcomes in these complex patients.

## Conclusions

Pediatric abdominal aortic aneurysms are exceptionally rare and differ fundamentally from adult aneurysms in etiology and management. This case highlights the association between tuberous sclerosis complex and clinically significant vascular abnormalities, underscoring the importance of comprehensive systemic screening in affected patients. Open surgical repair remains the primary treatment modality in infants, given the limitations of endovascular approaches.

Early postoperative graft thrombosis, although uncommon, may occur and necessitate vigilant perioperative monitoring, appropriate anticoagulation, and long-term clinical and imaging surveillance to ensure graft patency and optimize outcomes.
